# Dr. Edward Everett Hyde, A. B.

**Published:** 1913-08

**Authors:** 


Dr. Edward Everett Hyde, A. B., Knox College, 1896, M. D. P. & S. of Chicago, 1900, died July 4, aged 38, of acute myelogenic leucocythaemia. Shortly after receiving his medical degree he was ordained a minister of the Christian Church and went to the Caroline Islands as a missionary, returning the next year on account of his wife's health. Since 1902 he had served as assistant to the Editor of the Journal of the A. M. A., most acceptably to all. We knew him only by correspondence and one or two very brief meetings, but wish to acknowledge here his many courtesies. His death was undoubtedly hastened by his fatigue at the Minneapolis meeting of the A. M. A., where he had charge of the Daily Bulletin and discharged other arduous duties. The serious nature of the weakness which he felt was not recognized till after the accomplishment of this last task, a few days before his death. "Peace hath her heroes no less than war."

